# The Metabolic Syndrome, Oxidative Stress, Environment, and Cardiovascular Disease: The Great Exploration

**DOI:** 10.1155/2012/271028

**Published:** 2012-07-09

**Authors:** Rebecca Hutcheson, Petra Rocic

**Affiliations:** Department of Biochemistry and Molecular Biology, College of Medicine, University of South Alabama, 307 North University Boulevard, Mobile, AL 36688, USA

## Abstract

The metabolic syndrome affects 30% of the US population with increasing prevalence. In this paper, we explore the relationship between the metabolic syndrome and the incidence and severity of cardiovascular disease in general and coronary artery disease (CAD) in particular. Furthermore, we look at the impact of metabolic syndrome on outcomes of coronary revascularization therapies including CABG, PTCA, and coronary collateral development. We also examine the association between the metabolic syndrome and its individual component pathologies and oxidative stress. Related, we explore the interaction between the main external sources of oxidative stress, cigarette smoke and air pollution, and metabolic syndrome and the effect of this interaction on CAD. We discuss the apparent lack of positive effect of antioxidants on cardiovascular outcomes in large clinical trials with emphasis on some of the limitations of these trials. Finally, we present evidence for successful use of antioxidant properties of pharmacological agents, including metformin, statins, angiotensin II type I receptor blockers (ARBs), and angiotensin II converting enzyme (ACE) inhibitors, for prevention and treatment of the cardiovascular complications of the metabolic syndrome.

## 1. Introduction

Metabolic syndrome is a term that describes a cluster of independent risk factors that increase the likelihood of cardiovascular disease [1–3]. This syndrome affects as much as 30% of the United States population with increasing prevalence [4]. Although a consensus criterion has not been reached for diagnosing metabolic syndrome, it is recommended that screening should consider central obesity, insulin resistance, dyslipidemia (elevated triglycerides and low density lipoprotein (LDL) and decreased high density lipoprotein (HDL)), and high blood pressure [5]. Other factors such as proinflammatory and prothrombotic states have also been associated with metabolic syndrome [6]. 

Increased cardiovascular risk in the metabolic syndrome is the result of a complex interaction of the individual risk factors that is not fully understood. For example, although central obesity is a defining characteristic of the metabolic syndrome, a study of middle aged men with metabolic syndrome found that cardiovascular risk is also increased independently of body mass index with the metabolic syndrome [7]. Also, an association of increased risk of ischemic heart disease as well as ischemic stroke in metabolic syndrome was observed in a study of less obese metabolic syndrome patients [8]. Another study found that metabolic syndrome patients who also exhibit endothelial dysfunction are at a greater risk for cardiovascular disease than either group alone [3]. Thus, metabolic syndrome increases the likelihood of cardiovascular disease to an extent greater than the likelihood conferred by any of its individual components. 

Increased oxidative stress has emerged as playing a central role in metabolic syndrome and its component pathologies and may be a unifying factor in the progression of this disease. Reactive oxygen species (ROS) are highly reactive derivatives of oxygen metabolism. These short-lived molecules play important roles in normal physiological processes such as gene expression and signal transduction. In a healthy condition, ROS are maintained at an optimal level due to a balance between their production and elimination by enzymatic (superoxide dismutase, glutathione, catalase, peroxidase) and nonenzymatic (vitamins C and E) antioxidants. In a pathological state such as the metabolic syndrome, an increased oxidant capacity coupled with decreased antioxidant capacity creates an unbalanced environment that results in oxidative stress. Increased ROS levels manifested during oxidative stress have toxic effects on cells and tissues through increased oxidation of carbohydrates, lipids, and proteins. ROS have been shown to play a major role in the development and progression of cardiovascular disease [9–11]. Moreover, oxidative stress has been identified as a major mechanism of micro- and macrovascular complications in the metabolic syndrome [12]. 

## 2. Component Pathologies of the Metabolic ****Syndrome and Oxidative Stress 

### 2.1. Oxidative Stress is a Hallmark of the Metabolic Syndrome

Patients with metabolic syndrome often develop advanced atherosclerosis. Oxidative stress plays a central role in the initiation and progression of atherosclerosis. NAD(P)H oxidases are the primary source of ROS in the vasculature. Increased expression and activity of the phagocytic NAD(P)H oxidases with a parallel increase of oxidized LDL (oxLDL) and nitrotyrosine levels accompanied by thickened intima to media ratio in the carotid arteries, indicative of early subclinical atherosclerosis, have been demonstrated in metabolic syndrome patients [13]. It has also been found that subfractions of small HDL cholesterol particles, which are normally protective, posses lower antioxidant capacity in the metabolic syndrome [14]. Increased oxidative stress associated with increased production of ROS is augmented by decreased expression of antioxidant enzymes. Studies in a diet-induced rat model of metabolic syndrome found increased oxidative stress and endothelial dysfunction. This study further demonstrated increased ROS production capacity by the NAD(P)H oxidase along with downregulation of key superoxide dismutase (SOD) isoforms indicating a disrupted antioxidant defense system in metabolic syndrome [15]. Reports from the Third National Health and Nutrition Examination Survey indicate diminished concentrations of the antioxidants vitamins C and E and several carotenoids, even after adjusting for lower fruit and vegetable consumption in participants with metabolic syndrome [16]. Thus, it is clear that the human metabolic syndrome is characterized by oxidative stress precipitated by excess production of ROS and diminished antioxidant defenses. 

### 2.2. Oxidative Stress and Obesity

Recently, there has been some attempts to define the contribution of the individual components of the metabolic syndrome to oxidative stress evident in the metabolic syndrome patients. Obesity is a core component in the development of metabolic syndrome and plays a central role in amplified oxidative stress. Obese patients have shown oxidative stress-induced decreased vasodilatory response to acetylcholine, which was inversely related to body mass index, waste to hip ratio, fasting insulin, and insulin resistance [17]. Obesity in children, without any other metabolic syndrome components, has been repeatedly correlated with increased oxidative stress and endothelial dysfunction [18]. Weight loss (10% of body weight) by moderate diet restriction and moderate-intensity aerobic exercise in metabolic syndrome patients has been shown to improve markers of oxidative stress [19]. On the other hand, data from an intensive 21-day residential diet and exercise program in overweight or obese patients revealed a decrease in oxidative stress and improvement in other markers of cardiovascular risk asociated with metabolic syndrome even before significant weight loss [20]. This effect could have been mediated by a reduction in oxidative stress through exercise-mediated improvement in endothelial function and nitric oxide (NO) production or upregulation of antioxidant defenses. In animal models, ROS production in adipose tissue of obese mice was reduced by treatment with the NAD(P)H oxidase inhibitor apocynin resulting in improvement in glucose and lipid metabolism independent of body weight [21]. Long-term studies are needed to see if these short-term effects translate to long-term cardiovascular outcomes.

### 2.3. Oxidative Stress and Insulin Resistance

The isolated contribution of insulin resistance to oxidative stress is difficult to asscertain. Studies which address the question of oxidative stress in type II diabetes typically do not distinguish between the study participants on the basis of obesity or their lipid profile. Since both obesity and dyslipidemia, independently, significantly contribute to oxidative stress, and obesity is the primary risk factor for the development of insulin resistance, with dyslipidemia now emerging as a possible contributing factor, this presents a significant obstacle with respect to determining whether insulin resistance alone elevates oxidative stress in humans. Likewise, the animal models of insulin resistance are obese, and the insulin resistance develops secondary to obesity. Increased ROS have also been shown to have a causal role insulin resistance [22]. Both tumor necrosis factor *α* (TNF-*α*) and dexamethasone decreased Akt phosphorylation and consequently glucose uptake into cultured muscle cells, which was reversed by antioxidant treatment (N-acetyl cysteine (NAC), SOD, catalase, manganese (III) tetrakis (4-benzoic acid) porphyrin (MnTBAP)). The same study furthermore showed that glucose uptake was compromised in obese (db/db) mice in vivo resulting in increased blood glucose and antioxidants lowered blood glucose [22]. 

### 2.4. Oxidative Stress and Hyperglycemia

While hyperglycemia per se is not a defining parameter of the metabolic syndrome, hyperglycemia, which results from primary *β*-cell destruction in the absence of any other components of the metabolic syndrome, has been shown to correlate with elevated oxidative stress (decreased glutathione, GSH/GSSG ratio) in type I diabetes [23]. However, this may or may not be relevant to the metabolic syndrome, where hyperglycemia develops secondary to the development of insulin resistance. 

### 2.5. Oxidative Stress and Dyslipidemia

Dyslipidemia, characterized by elevated LDL and triglycerides and decreased HDL, is also a frequent component of the metabolic syndrome phenotype. A positive correlation between elevated LDL and triglycerides and low HDL and oxidative stress in animal models is well established. LDL receptor-deficient mice fed a cholesterol-enriched diet developed elevated LDL levels and consequently oxidative stress [24]. These observations extend to human studies. High plasma oxidative stress markers positively correlated with elevated plasma triglycerides and inversely correlated with low HDL [25] in a group of metabolic syndrome patients with end-stage renal disease, after all other factors (presence of obesity, hypertension, and/or type II diabetes) were adjusted for. Lipid peroxidation, as an index of oxidative stress, correlated with low HDL levels, irrespective of age, gender, and presence of the other metabolic syndrome components [26]. It is also now accepted that the numerous positive effects of some statins in the cardiovascular system are independent of their lipid-lowering effect and a consequence of a direct decrease in oxidative stress. For example, short-term pravastatin treatment reduced myocardial infarct (MI) size in hypercholesterolemic rabbits through reduction in peroxynitrate and nitrotyrosine formation [27]. Similar results, with regards to the atherogenic index, were achieved with rosuvastatin, which lowered oxidative stress by elevating the expression of antioxidant enzymes (SOD, catalase, glutathione, glutathione peroxidase), LDL, triglycerides, and C-reactive protein (CRP) and elevated HDL [28]. 

### 2.6. Oxidative Stress and Hypertension

Hypertension is another component of the metabolic syndrome which is independently associated with increased cardiovascular risk. While animal models of hypertension have also been rather consistently associated with elevated oxidative stress, whether hypertension alone increases oxidative stress in humans is somewhat controversial. One study found no difference in markers of oxidative stress when comparing hypertensive and normotensive patients [29], while several studies found increased oxidative stress in hypertensive patients [30, 31]. A study in metabolic syndrome patients showed that other metabolic syndrome components (low HDL, triglycerides, abdominal obesity, and fasting glucose) had minimal contribution to increased oxidative stress, whereas hypertension alone was responsible for elevated oxidative stress in these patients [32]. This study, however, used the International Diabetes Federation (IDF) definition for metabolic syndrome, which uses body mass index (BMI) as an indicator of central obesity and has been criticized for underdiagnosing metabolic syndrome patients [33]. The Adult Treatment Panel (ATP) III metabolic syndrome definition, which uses waist circumference as a measurement of central obesity, has been shown to be a better predictor of mortality than the IDF definition [34]. It is not clear how the effects of hypertension were separated from the effects of the other risk factors in the established pathology of the metabolic syndrome in this study. In another study showing a positive link between hypertension and oxidative stress, seemingly essential hypertension was found to, in fact, be secondary to insulin resistance [35]. Another study determined that CRP, an inflammatory marker, known to be elevated in metabolic syndrome, is a better predictor of oxidative stress in “essential” hypertension, than high blood pressure [36]. These studies illustrate that attempts to identify the etiology of oxidative stress in human metabolic syndrome where hypertension is a component are complicated by the propensity of additional metabolic syndrome components to complicate the interpretation of the study results as confounding factors so that the isolated contribution of hypertension to oxidative stress becomes difficult to determine. 

Furthermore, unlike the other component pathologies of the metabolic syndrome, hypertension is itself a multifactorial disease with a variety of possible etiologies. Oxidative stress has been shown to increase deoxycorticosterone acetate- (DOCA-) salt [37], angiotensin II-(Ang II-)-infusion [38] and 2-kidney-1-clip-induced [39] as well as in genetic animal models of spontaneous hypertension (SHR). However, norepinephrine-induced hypertension does not increase oxidative stress in a rat model [40]. These studies may indicate that whether or not human hypertension is associated with oxidative stress depends on the predominant etiology of the disease in the individual patient. This may explain the seeming discrepancy among the studies outlined above. 

### 2.7. Lessons from Children with Metabolic Syndrome

Although a definition of the metabolic syndrome in children has not been agreed upon, development of characteristics of metabolic syndrome is increasingly prevalent in children and adolescents. Childhood obesity has been associated with development of cardiovascular risk factors [41]. Autopsies of young people revealed that increased number of cardiovascular risk factors results in increased severity of coronary and aortic atherosclerosis [42]. A swine model of obesity showed that early obesity is associated with vascular oxidative stress and endothelial dysfunction even before development of insulin resistance or systemic oxidative stress [43]. This was confirmed in obese children, where obesity, without any other metabolic syndrome components, has been repeatedly correlated with increased oxidative stress and endothelial dysfunction [18]. Additional components of metabolic syndrome further increased oxidative stress in overweight children [44], perhaps indicating that compounding the component pathologies of the metabolic syndrome multiplies oxidative stress by some as yet undetermined factor.

## 3. Interactions of Metabolic Syndrome ****and Its Individual Component Pathologies ****with Environment and Lifestyle Factors

Cigarette smoke and air pollution are the most significant external sources of oxidative stress. Epidemiological studies have demonstrated a clear association between increased air pollution and human morbidity and mortality. Production of ROS is the fundamental mechanism which mediates these detrimental effects [45]. Short-term exposure to urban air pollution in healthy young adults resulted in increased oxidative stress that is not confined within the lungs [46]. The main arbitrators of air pollution-derived effects are aromatic hydrocarbon and metal-containing inhalable nanoparticles which can penetrate the alveolar-septal barrier and thus generate oxidative stress both via activation of alveolar macrophage and systemic vascular oxidases including the NAD(P)H, mitochondrial and xanthine oxidases [47, 48]. Aromatic hydrocarbons generate ROS through redox cycling of quinone-based radicals, by complexing of metals resulting in increased electron transport and by depletion of antioxidants by reactions between quinones and thiol-containing compounds. Metals directly support electron transport to generate oxidants and also diminish levels of antioxidants [45]. In addition to direct generation of ROS, cellular responses to oxidative stress after nanoparticle exposure contribute to the overall damage. Oxidative stress initiates activation of proapoptotic signal transduction cascades and release of inflammatory mediators, which ultimately lead to cell death, especially of endothelial cells. Endothelial cell damage and death is a key event in the development and worsening of CAD and other vascular pathologies. 

### 3.1. Cardiovascular Consequences of Cigarette Smoke: Impact of the Metabolic Syndrome

Smoking and air pollution interact with the metabolic syndrome in ways which are as yet insufficiently understood but clearly combine to deliver a cardiovascular risk factor which is greater than the sum of its parts. Smokers and ex-smokers are more likely to have metabolic syndrome than nonsmokers [49]. Current smokers have even higher rates of both metabolic syndrome and its individual components than nonsmokers [50]. Both cigarette smoking and the metabolic syndrome are strong independent risk factors for cardiovascular disease; however, smoking also potentiates the negative cardiovascular effects of the metabolic syndrome. For example, metabolic syndrome is associated with higher rates of cardiac events after acute myocardial infarction and smoking has an additive effect [51]. These effects are largely mediated via ROS generation [52]. 

### 3.2. Cardiovascular Consequences of Environmental Pollution: Impact of the Metabolic Syndrome

Secondary to numerous studies having reported similar effects [53, 54], a scientific statement issued by the American Heart Association in 2010 implicated particulate matter air pollution as a trigger for cardiovascular disease. Studies revealed increased cardiovascular risk in both short- and long-term exposure with higher particulate matter air pollution related mortality risk for cardiovascular than for pulmonary diseases. Air pollution has been associated with an increased risk of myocardial infarction [55]. Obese people may be at an increased risk [56]. In Sao Paulo, Brazil, cardiovascular disease emergency room visits were 20% higher in patients with type II diabetes than in nondiabetics indicating that diabetics may be more susceptible to the adverse effects of air pollution [57]. It is unclear whether the patients could be classified as metabolic syndrome or not. Another study reported a 2-fold increased risk of MI in diabetic patients versus non-diabetics exposed to the same amount of environmental pollution [58]. In both studies, end-points (emergency room visits and incidence of MI) were normalized to population statistics in areas of lesser air pollution. Nanoparticle and carbon monoxide air pollution elicited autonomic nervous system dysfunction, which manifested in significant heart rate variations in metabolic syndrome but not in normal subjects [59]. 

Moreover, studies in animal models and humans suggest that long-term exposure to environmental pollutants promotes development of insulin resistance, hyperglycemia, hypertension, obesity and the metabolic syndrome. Workers in refineries and residents in surrounding areas have been found to have high incidence of the metabolic syndrome [60–62]. Benzene derivatives, major byproducts of petrochemical reactions, induce hyperinsulinemia in a dose-dependent manner in animal studies, which could provide a mechanism of development of insulin resistance in the metabolic syndrome. Bisphenol A, an essential ingredient in plastic polymer production found in significant quantities in the urine of 95% of the US population, at doses 1000-fold less than those allowed by the Environmental Protection Agency (EPA) decreases glucose tolerance and induces insulin resistance [63] by decreasing glucose transporter 4 (GLUT-4) expression [64]. Long-term exposure to lead has been repeatedly correlated with hypertension [65]. Subtoxic levels of arsenic in drinking water have been correlated with high prevalence of type II diabetes in numerous studies across the world [66–69]. Arsenic inhibits Akt phosphorylation [70], an event critical for GLUT-4 transporter translocation to the membrane and glucose uptake. A study in mice found that long-term exposure to air pollutants may promote development of insulin resistance, obesity, and metabolic syndrome [71]. Therefore, not only do air pollution and environmental toxins exacerbate cardiovascular complications in patients with existing metabolic syndrome but they also promote the development of the metabolic syndrome.

### 3.3. Effect of Diet on Oxidative Stress in the Metabolic Syndrome

Several studies have investigated a role for dietary influence on oxidative status. Mediterranean-style diet intervention consisting of increased intake of whole grains, fruits, vegetables, nuts, and olive oil for two years resulted in decreased CRP levels as well as improved insulin resistance and endothelial function [72]. CRP levels have been shown to be increased by increased oxidative stress [73, 74]. The beneficial effects of the Mediterranean diet are further supported by findings that increased consumption of virgin olive oil improved antioxidant status with decreased oxidative stress [75]. In contrast, The Oxford Fruit and Vegetable Study Group reported only a small increase in antioxidant concentration with associated decrease in blood pressure with increased consumption of fruit and vegetables in the diet of healthy subjects [76] suggesting a greater benefit of dietary interventions for the metabolic syndrome population. Green tea supplementation reduced body weight and BMI and had a beneficial effect on lipid peroxidation in obese metabolic syndrome patients [77]. Results from a recent study support that adequate dietary intake of dairy results in improvement in markers of oxidative stress in metabolic syndrome [78]. 

## 4. Impact of Metabolic Syndrome and Its ****Individual Component Pathologies on ****Severity of Cardiovascular Disease 

### 4.1. Impact of the Metabolic Syndrome on Incidence and Severity of Coronary Artery Disease

Metabolic syndrome patients have a significantly greater risk for the development of cardiovascular disease in general and coronary artery disease (CAD) in particular. Several studies report a correlation between metabolic syndrome and carotid atherosclerosis [79]. The clustering of abdominal obesity with 2 or more component pathologies of the metabolic syndrome without hyperglycemia resulted in an ~2.5 times (range 1.45–6.22) higher incidence of elevated carotid intima-media thickness, an early indicator of subclinical atherosclerosis, while in those with hyperglycemia the incidence was ~6 times (range 2.64–11.8) higher [80]. Elevated blood glucose on the background of abdominal obesity strongly correlated with CAD development in women, while low HDL on the background of abdominal obesity was a stronger predictor for CAD development and severity in men [81]. Even abdominal obesity alone, without additional metabolic syndrome components, seems to predict future cardiovascular risk in men but not in women [82]. In addition to increased incidence of CAD, the metabolic syndrome is associated with more severe ischemic CAD, and a higher number of the metabolic syndrome components have been correlated with worse CAD by coronary angiography [83, 84]. Patients with insulin resistance and hyperglycemia are ~2 times more likely to die of CAD than patients with CAD but without insulin resistance or hyperglycemia. Patients with all component pathologies of the metabolic syndrome are ~3.6–4.4 times more likely to die of CAD [85, 86]. 

The etiology for these phenomena may be related to elevated oxidative stress in the metabolic syndrome. Increased oxidative stress has been strongly associated with atherosclerosis leading to CAD [87]. In fact, a specific element essential in the initiation of atherosclerosis, oxLDL, has emerged as the single strongest predictor of CAD compared with the conventional lipoprotein profile (LDL, HDL, triglycerides) and other traditional risk factors (BMI or waist circumference, individual component pathologies of the metabolic syndrome or metabolic syndrome, smoking). Elevated oxLDL confers a 4.25 greater probability of CAD development [88] and has been found to directly correlate with HDL levels but, interestingly, to be independent of any other components of the metabolic syndrome as well as age, gender, and inflammatory markers [26]. Thus, elevated oxLDL confers a similar risk to that imparted by the metabolic syndrome but not by any of its individual components. 

### 4.2. Impact of the Metabolic Syndrome on Outcomes of Treatments for CAD: PTCA and CABG

In addition to more severe CAD with worse long-term prognosis, current revascualrization therapies, coronary artery bypass grafting (CABG), and percutaneous transluminal coronary angioplasty (PTCA) in metabolic syndrome patients are associated with higher procedural risk and poorer long-term outcomes [89–91]. In one study of the 551 metabolic syndrome patients who underwent coronary revascualrization by either CABG or PTCA, 256 underwent revascualrization within 10 years, and 221 died within that time period (118 due to cardiovascular events) [92]. Metabolic syndrome patients have been shown to have an increased inflammatory response following PTCA than both healthy patients and patients with diabetes mellitus [93]. In a study in which patients were followed for 4 years after PTCA using sirolimus-eluting stents, incidence of in-stent thrombosis after PTCA was comparable between metabolic syndrome patients without insulin resistance or hyperglycemia and patients without metabolic syndrome (0.6% and 0.3%, respectively); however, annual mortality rates were 3 times higher in the metabolic syndrome patients (3%). In metabolic syndrome patients with insulin resistance and hyperglycemia, in-stent thrombosis was 6 times higher (6.1%) and annual mortality 5 times higher (5.6%) [94]. Following CABG, metabolic syndrome patients have an increased incidence of adverse cardiac events and re-appearance of angiographically significant lesions in 2 or more vessels, due to either graft failure or new lesion formarton, within 2–5 years. This effect appears to correlate closely with elevated triglycerides and blood glucose [95–97]. A recent study based on data from the Cleveland Clinic over the last 20 years found HDL levels to be the most important predictor of survival in post-CABG patients [98]. This is interesting in light of low HDL being the only parameter which strongly correlated with elevated oxLDL, which also appears to most accurately predict CAD risk development. 

### 4.3. Impact of the Metabolic Syndrome on Outcomes of Treatments for CAD: Coronary Collateral Growth 

With the limited effectiveness of the current treatments for occlusive CAD in the metabolic syndrome patient population, significant effort has been aimed at developing alternative means for coronary revascualrization. Narrowing of the coronary arteries due to accumulation of atherosclerotic plaque leads to decrease in blood flow to distal tissue. In response to increased myocardial oxygen demand, heart tissue distal to the occlusion undergoes transient, repetitive ischemia (RI) as in stable angina pectoris. The physiological response of the heart is to enlarge native collateral arterioles to conduit vessels in a process termed coronary collateral growth or arteriogenesis [99]. This protects the heart from ischemic damage by restoring blood supply to heart tissue distal to the occluded artery. However, the ability to enlarge native collaterals is impaired in metabolic syndrome patients [100]. Yilmaz et al. showed that the prevalence of type II diabetes and the metabolic syndrome were higher in patients exhibiting poor coronary collateral development than those exhibiting good coronary collaterals (44% (diabetes), 78.4% (metabolic syndrome) versus 27.1% (diabetes), 49.2% (metabolic syndrome)). The metabolic syndrome remained an independent risk factor for poor coronary collaterals even after adjusting for type II diabetes [100]. The number or type of metabolic syndrome components other than diabetes was not differentiated in this study. Sasmaz and Yilmaz showed that an increasing number of component pathologies of the metabolic syndrome correlated with increasingly poorer coronary collateral development by angiography using the Cohen and Rentrop grading systems [101]. Mouquet et al. also found that increasing the number of component pathologies of the metabolic syndrome inversely correlated with coronary collateral development by angiographic grading. In addition, they determined that of the individual components of the metabolic syndrome hyperglycemia, hypertension and insulin resistance negatively correlated with coronary collateral development, with hyperglycemia having the strongest negative correlation and insulin resistance the weakest [102]. Thus, restoration of coronary collateral growth is a potential noninvasive strategy for treating occlusive CAD in this patient population. 

Studies in animal models of diabetes and the metabolic syndrome support the findings in humans. Coronary collateral growth in response to coronary artery occlusion has been shown to be impaired in rat models of the metabolic syndrome [103, 104] and a dog model of dextrose infusion [105]. However, normal collateral development has been reported in a swine model of the metabolic syndrome [106]. The most obvious difference between the rat and dog models and the swine model is that the studies in the rat and dog models used transient, repetitive coronary artery occlusion to stimulate collateral development, which mimics the situation in the human, whereas the swine model is a model of progressive chronic ischemia. Since the exact duration of coronary occlusions has been associated with the extent of collateral growth [107, 108], this difference between the two animal models is the likely explanation for the different outcomes between the rat and dog versus the swine models.

Oxidative stress is emerging as a major underlying mechanism of impaired collateral growth in the metabolic syndrome. It has now been clear for several years that an optimal amount of ROS or an optimal redox state of the cell (redox window) is absolutely required for coronary collateral growth. This topic was recently extensively reviewed [99]. Briefly, our own and Chilian's group have demonstrated that reduction of ROS below the lower boundary of this window reduces collateral growth but increasing ROS above the upper boundary of this window is likewise incompatible with collateral development [99, 103, 109]. Either decreasing superoxide (O_2_^−•^) with a flavin-containing oxidase inhibitor (diphenyleneiodonium (DPI)) or increasing O_2_^−•^ with an SOD inhibitor (diethyldithiocarbamic acid (DETC)) abrogated coronary collateral growth in normal, healthy rats [109]. Furthermore, decreasing oxidative stress by apocynin or Ang II type I receptor blockade in normal rats impaired coronary collateral growth, but significantly improved coronary collateral growth in the metabolic syndrome rat model where basal and repetitive occlusion-induced oxidative stress is elevated [103]. Thus, in normal, healthy animals, the amount of ROS generated by repetitive coronary occlusion is necessary for coronary collateral development. However, in the metabolic syndrome animals where baseline levels of ROS are elevated, the amount of ROS generated by repetitive coronary occlusion is much higher and is not compatible with coronary collateral development [99]. This mechanism might underlie the impaired coronary collateral development in the metabolic syndrome patients.

Of the possible sources of ROS, the sources most important for the regulation of coronary collateral growth have not yet been entirely resolved. Strong evidence now points to the mitochondrial sources of ROS. In a recent study, the mitochondria-targeted antioxidant MitoQ nearly completely restored coronary collateral growth in a rat model of the metabolic syndrome, the Zucker obese fatty rat (ZOF) [110]. Several studies suggest that membrane NAD(P)H oxidases are also important sources of ROS within the context of collateral growth [111, 112]. Whether the crosstalk between membrane NAD(P)H oxidases and the mitochondria, phenomenon known as ROS-induced ROS release, is functionally relevant in collateral growth remains to be determined.

## 5. Effect of Antioxidant Therapies on the ****Metabolic Syndrome and Its Individual ****Component Pathologies

### 5.1. Lessons from Antioxidant Clinical Trials

Results from clinical trials for improving cardiovascular outcomes by antioxidant therapy have, however, been inconsistent and confusing. Antioxidant supplementation in humans has not been as successful as expected although some studies have been promising. The HOPE and HOPE-TOO clinical trials evaluated long-term vitamin E therapy in patients at least 55 years old who had either vascular disease or diabetes mellitus. There was no improvement in cardiovascular outcomes. Alarmingly, there was an increase in heart failure and heart-failure-related hospitalizations [113]. Similar results were obtained in the MRC/BHF Heart Protection study. In contrast, a pooled analysis of nine cohort studies found that vitamin C but not vitamin E reduced incidence of major coronary heart disease [114]. 

However, multiple factors complicate the interpretation of the results of these trials. First, whether the antioxidant interventions actually succeeded in reducing oxidative stress in patients enrolled in the HOPE and the MRC/BHF trials was never ascertained [115]. Since many of the patients enrolled in these trials were already on drugs with known oxidative stress lowering effects, including angiotensin converting enzyme (ACE) inhibitors or Ang II type I receptor inhibitors (ARBs), metformin and statins, it is probable that there was in fact no additional effect of antioxidants on ROS levels. In support of this proposition, vitamin E failed to lower oxidative stress in double-blind studies in healthy individuals with intact antioxidant defenses demonstrating that antioxidants are ineffective under conditions where there is no oxidative stress [116]. Moreover, in animal studies, treatment with antioxidants decreased ROS levels and improved coronary collateral growth in metabolic syndrome animals with elevated basal oxidative stress, but actually decreased coronary collateral growth in healthy animals with no evidence of basal oxidative stress [111], indicating that administering antioxidants on the background of normal ROS levels does not confer a beneficial cardiovascular effect. Thus, antioxidant supplementation does not reduce the risk of developing metabolic syndrome in healthy subjects [117], and lack of cardiovascular benefits found in the large scale clinical trials may not be representative of untreated metabolic syndrome patients. However, supplementation may improve cardiovascular risk in patients with established metabolic syndrome as these are patients with a decreased antioxidant capacity [118, 119]. In metabolic syndrome patients, infusion of vitamin C decreased oxidative stress markers and improved arterial flow-mediated dilation [120]. Daily cranberry juice for eight weeks increased antioxidant capacity and reduced lipid oxidation in metabolic syndrome women [121]. 

 Second, the effectiveness of antioxidants used in clinical trials is low. Both vitamins E and C have actually been shown to have some prooxidant effects in vitro [122, 123] and are, at the doses administered in the clinical trials, unlikely to affect plasma or tissue ROS levels [124]. In addition, vitamin E does not inhibit some significant elements of ROS-induced damage in the metabolic syndrome, for example, myeloperoxidase-induced lipid peroxidation [125]. Specifically with respect to ischemic heart disease, emerging evidence suggests that reduction in mitochondrial oxidative stress may be critical for myocardial adaptations to ischemia, including collateral growth and other aspects of ischemic preconditioning; the antioxidants in these trials were not targeted to the mitochondria and therefore could not have reduced mitochondrial oxidative stress. Also, collateral growth and myocardial perfusion per se were not the end-points in these trials; therefore, a multitude of additional factors, most probably heart failure, contributed to total outcomes. 

### 5.2. Antioxidant Properties of Metformin, Statins, ACE Inhibitors, and ARBs

 The beneficial effect of lowering oxidative stress on cardiovascular outcomes in metabolic syndrome patients can perhaps be further supported by beneficial effects of the drugs typically used to treat the metabolic syndrome and/or its various components, specifically metformin, statins, ACE inhibitors, and ARBs. All of these pharmacological agents have been found to have beneficial cardiovascular effects independent of their original purpose, that is, glycemic control (metformin), lipid lowering (statins), and blood pressure regulation (ACE inhibitors and ARBs). These beneficial cardiovascular effects may be mediated by their antioxidant properties. Metformin has been shown to decrease intracellular ROS by upregulating thioredoxin in cell culture [126]. In human umbilical vein endothelial cell (HUVEC) culture, metformin inhibited advanced glycation end-product-(AGE-) induced ROS formation [127], also suggesting a possibility that its protective effects on the vasculature are mediated via its direct antioxidant effects. In the rat kidney, metformin increased antioxidant defenses by upregulating catalase and glutathione, which accounted for significant protection against diabetic nephropathy [128]. In a clinical study, metformin decreased carotid intima-media thickness, plasma indexes of inflammation and oxidative stress, and arterial stiffness in a group of metabolic syndrome patients [129]. 

It is also now accepted that the numerous positive effects of some statins in the cardiovascular system are mediated independently of their lipid-lowering effect via a direct decrease in oxidative stress. As mentioned earlier in this paper, short-term pravastatin treatment reduced MI size in hypercholesterolemic rabbits through reduction in peroxynitrate and nitrotyrosine formation [27]. Similar results, with regards to the atherogenic index, were achieved with rosuvastatin, which lowered oxidative stress by elevating the expression of antioxidant enzymes, superoxide dismutase, catalase, glutathione, and glutathione peroxidase in addition to lowering LDL, triglycerides, and CRP and elevating HDL [28].

Several clinical trials have documented beneficial effects of ACE inhibitors and ARBs on cardiovascular end-points in type II diabetic and metabolic syndrome patients without hypertension. The HOPE study showed a 22% reduction in cardiovascular events (MI, stroke, cardiac arrest, revascualrization, heart failure, death) in metabolic syndrome patients and without hypertension treated with an ACE inhibitor, ramipril versus metabolic syndrome patients without hypertension not treated with an ACE inhibitor [130]. Nearly identical results were achieved with an ARB, telmisartan (ONTARGET trial) [131]. Ang II is a potent generator of vascular and myocardial ROS through the activation of NAD(P)H oxidases [132, 133], and consequently likely mitochondrial ROS generation via the phenomenon of ROS-induced ROS release. ACE inhibitors and ARBs have been shown to downregulate ROS in cell culture and in vivo, with ARBs, although much less frequently used, showing a statistically significantly greater effect. Losartan reduced oxidative stress generation and development of pressure overload-induced left ventricular hypertrophy in a rat model [134]. An ACE inhibitor, quinapril, reduced plasma markers of oxidative stress in metabolic syndrome patients [135]. Olmesartan, today's most frequently used ARB for patients at risk for CAD development due to its strong anti-inflammatory properties, added to an ACE inhibitor resulted in a greater decrease in oxidative stress and a significant improvement in cardiac function in advanced diastolic heart failure in hypertensive patients [136].

In addition to lowering oxidative stress, Ang II blockade has been shown to have marked positive effects on insulin resistance, glucose tolerance, and the lipid profile. In a rat model of insulin resistance and renin-angiotensin system (RAS) overactivity, the TG(mREN2)27 rat, administration of an ARB improved insulin sensitivity, stimulated glucose transport into muscle, and reduced oxidative stress [137]. ARBs likewise reduced insulin resistance in the obese and insulin resistant ZOF rats by increasing GLUT-4 transporters and glucose uptake [138]. Post-hoc analysis of the HOPE trial demonstrated a 32% reduction in the incidence of development of new-onset diabetes (insulin resistance and hyperglycemia) in patients treated with the ACE inhibitor, ramapril. Another study reported similar results in ZOF rats, where not only insulin and glucose but all metabolic parameters including LDL, HLD, and triglycerides as well as oxidative stress and vascular dysfunction were significantly improved in response to ACE inhibition (enapril); however, these parameters were not improved nearly as much in the Zucker diabetic fatty rat (ZDF), indicating that overt hyperglycemia is more resistant to Ang II inhibition [139]. A clinical study also showed a significant reduction not only in fasting blood glucose but also in LDL cholesterol and an increase in HDL cholesterol following 6 months of ARB or ACE inhibitor (losartan or enapril) treatment [140]. 

These effects are likely also indirectly mediated through the Ang II-generated oxidative stress, since Ang II has been shown to inhibit Akt phosphorylation and, consequently, GLUT-4 transporter translocation to the plasma membrane in an NAD(P)H oxidase-dependent manner via tyrosine nitration, probably through formation of peroxynitrate [141]. In fact another study has demonstrated that Ang II impairs insulin signaling and GLUT-4 translocation to the membrane in muscle fibers via generation of ROS, which could be reversed by ARBs or antioxidant treatment [142]. Therefore, the positive effects of ACE inhibitors and ARBs in the cardiovascular system, apart from lowering blood pressure thereby reducing hypertrophic vascular remodeling and afterload on the heart, can likely be attributed to their direct antioxidant effects as well as reduction in blood glucose and associated benefits, most notably reduction in AGEs and associated vascular remodeling (decreased compliance) and improvement in the lipid profile, specifically HDL levels, which tend to correlate with oxLDL, apparently the most predictive factor for CAD development.

## 6. Conclusions and Perspectives

In conclusion, it is clear that metabolic syndrome is associated with increased oxidative stress. Furthermore, it appears that some component pathologies of the metabolic syndrome contribute to a higher percentage of total oxidative stress than others; however, additional studies are needed to determine the exact contribution of individual components to total oxidative stress. 

It is also clear that the metabolic syndrome is a strong risk factor for the development and increased severity of cardiovascular disease in general and occlusive CAD in particular and confers a greater risk than the sum of its individual components. However, the presence of which individual component or what exact combination of individual components confers the greatest risk for CAD development remains a matter of debate and may be gender-specific with abdominal obesity in combination with low HDL and elevated oxLDL conffering the greatest risk for men, while hyperglycemia provides the greatest risk factor for women. Moreover, metabolic syndrome is a predictor of higher procedural risk and poorer postprocedure outcomes for revascualrization therapies, PTCA, and CABG, with insulin resistance and hyperglycemia confering the greatest negative effect. Finally, development of coronary collaterals is also severely compromised in the metabolic syndrome. Although the exact contribution of individual pathologies of the metabolic syndrome to oxidative stress is difficult to conclusively determine, it is certain that oxidative stress is highly elevated in the metabolic syndrome. We believe that ample evidence points to this increased oxidative stress being the major unifying mechanism which underlies the increased propensity for CAD development, greater severity of CAD at a younger age, and poorer treatment outcomes.

Air pollution and cigarette smoke pose a greater risk of adverse cardiovascular events for people with the metabolic syndrome possibly because of the increased oxidative stress in the metabolic syndrome, which is further elevated by the aromatic hydrocarbon and metal nanoparticle components of these environmental pollutants leading to activation of well-known detrimental cascades of events that link oxidative stress to exascerbation of cardiovascular disease. 

Finally, we would like to emphasize that despite the reported lack of success of large antioxidant trials, we believe that antioxidants might be useful for treatment and prevention of cardiovascular disease in metabolic syndrome patients. Several lines of evidence support this opinion. First, the drugs currently used to successfully retard the progression of cardiovascular and renal disease in patients with the metabolic syndrome all have strong direct antioxidant effects. Second, the effect of antioxidants on cardiovascular indexes was significant in metabolic syndrome patients in carefully designed studies where oxidative stress was in fact lowered. We suggest that experiences to date speak to the necessity of conducting well-designed large-scale trials which would include carefully selected populations of metabolic syndrome patients. On a related note, it is obvious that the metabolic syndrome is a distinct and complex phenotype with a set of as yet incompletely understood interactions which presents a unique set of challenges. Thus, cardiovascular disease in the metabolic syndrome cannot be adequately studied in a healthy animal model or in animal models which represent one of its component pathologies. It is therefore critical that therapeeutic endeavors aimed at resolution of CAD in the metabolic syndrome, including coronary revascualrization, be studied in animal models of the metabolic syndrome.
